# Assessing delayed penicillin hypersensitivity using the PENFAST+ score

**DOI:** 10.3389/falgy.2023.1302567

**Published:** 2023-11-13

**Authors:** Julie Castagna, François Chasset, Jean-Eric Autegarden, Claire Le Thai, Emmanuelle Amsler, Annick Barbaud, Angèle Soria

**Affiliations:** ^1^ Médecine Sorbonne Université, Service de dermatologie et d’allergologie, Hôpital Tenon, Assistance Publique des Hôpitaux de Paris (AP-HP), Paris, France; ^2^ Centre d’Immunologie et de Maladies Infectieuses, Cimi-Paris, INSERM, Paris, France; ^3^ INSERM, Institut Pierre Louis d’Epidémiologie et de Sante Publique, Paris, France

**Keywords:** penicillin allergy, diagnostic score, drug hypersensitivity, delayed hypersensitivity, PEN-FAST clinical decision rule, delabelling

## Abstract

**Introduction:**

Approximately 10% of individuals report a suspected allergy to penicillin, but according to allergy work-ups, only 10%–15% of them are truly allergic. A clinical decision score, the PEN-FAST, was developed and validated to identify adults with low-risk penicillin allergy.

**Objectives:**

The objective of this study was to improve the performance of the PEN-FAST score, particularly for those with delayed hypersensitivity (HS), by improving the negative predictive value.

**Methods:**

STEP 1: Retrospective evaluation of the PEN-FAST score in patients with proven immediate and delayed penicillin allergy. STEP 2: Identification of additional criteria among Step 1 patients misclassified by PEN-FAST score. Development of the PEN-FAST+ score using multivariable logistic regression in a prospective cohort of patients with a suspicion of HS to penicillin. STEP 3: Comparison of diagnostic performances of PEN-FAST and PEN-FAST+ scores.

**Results:**

The PEN-FAST score showed limitations in predicting the relapse of immediate skin HS or delayed maculopapular exanthema, with 28.6% and 38.4% of patients misclassified, respectively. We identified two potential additional criteria: skin rash lasting more than 7 days and immediate reaction occurring in less than 1 h (generalized or localized on palmoplantar area or scalp itching/heat feeling). A total of 32/252 (12.7%) patients were confirmed to be allergic to penicillin. With PEN-FAST, 37% of patients (*n* = 10) with delayed allergic penicillin HS were misclassified. With PEN-FAST+, 3 patients with delayed HS confirmed by a ST (11.1%) were misclassified. The AUC was significantly higher for PEN-FAST+ than PEN-FAST (85% vs. 72%, *p* = 0.03).

## Introduction

Approximately 10% of individuals report a suspected allergy to penicillin, but according to allergy work-ups, only 10% to 15% of them are truly allergic (1). Skin tests (STs) followed by a drug provocation test (DPT) with penicillin are the gold standard detection methods but are time-consuming (1). Trubiano *et al*. developed and validated a clinical decision score, the PEN-FAST, with 3 criteria (Five years or less since reaction [yes +2], Anaphylaxis or angioedema OR Severe cutaneous adverse reaction [yes +2], Treatment required for reaction (including unknown) [yes +1]), to identify adults with low-risk penicillin allergy (2).

A PEN-FAST score below 3 points is in favor of a low risk of allergy to penicillin. A score of less than 3 includes both patients with a PEN-FAST score of 0 and those with a PEN-FAST score of 1 or 2 with a very low (<1%) and low (5%), respectively, risk of having a positive penicillin allergy test. This score was validated in European and North American cohorts of adults with predominantly immediate hypersensitivity (HS). However, delayed HS, and more specifically maculopapular exanthema (MPE), represents the most common form of penicillin allergy (3, 4), whereas the percentage of patients with delayed HS represented 33.1% of the initial cohort (2).

Our first objective was to evaluate the decision score of penicillin allergy PEN-FAST in a retrospective cohort of patients with a proven allergy (immediate and delayed allergy to penicillin, especially in those with MPE). In these patients, the allergy to penicillin was shown after immediate or delayed reading of positive skin tests (STs).

The secondary objective was to improve the performance of the PEN-FAST score, particularly the negative predictive value (NPV). Challenging a patient with a penicillin allergy using the same or another penicillin is considered unjustifiable without STs for some authors, due to the potential risk of severity (5, 6). PEN-FAST has been validated in a prospective cohort, demonstrating non-inferiority compared to STs when direct drug provocation tests (DPT) were performed without STs if PEN-FAST scores were less than 3 before DPT. However, it is important to note that this analysis was limited to patients with immediate HS after DPT (7). It appears crucial to increase the probability of correctly classifying nonallergic patients, including those with delayed HS.

## Methods

**Figure 1:**
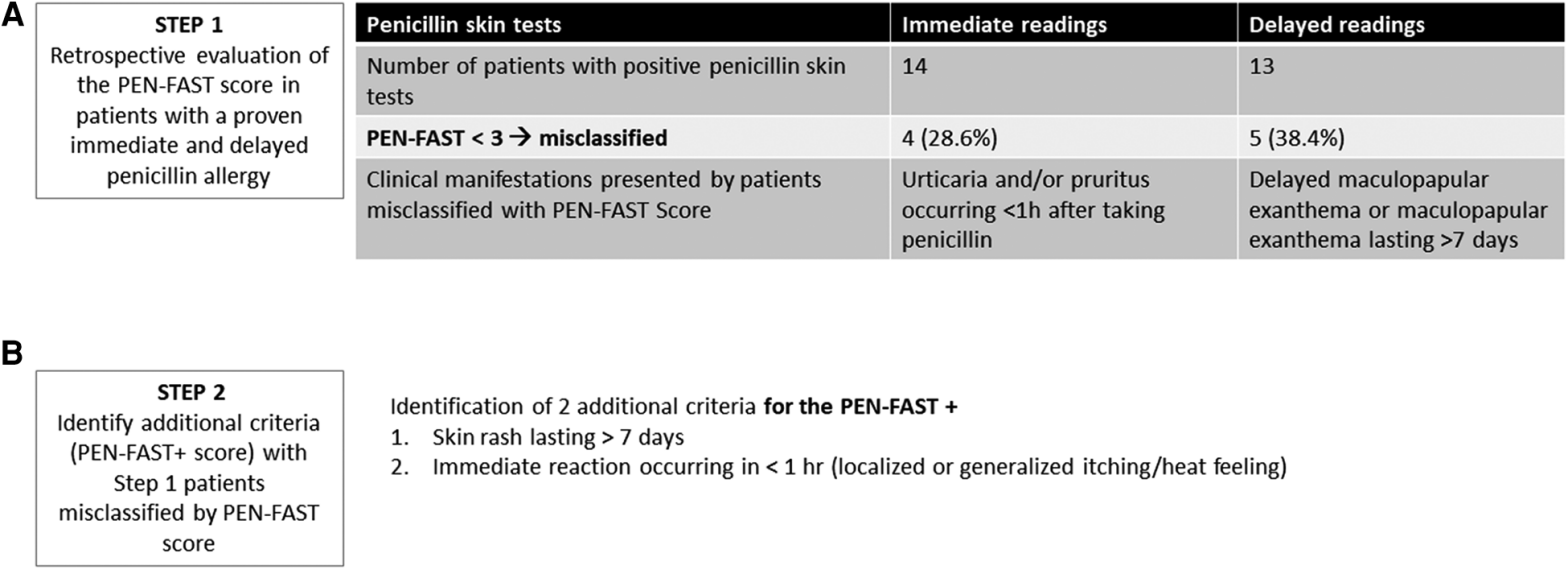
STEP 1 and 2 of the study. (**A**) Results of the analysis of PEN-FAST in 27 adults with true penicillin-allergy immediate and delayed. STs, skin. (**B**) Two additional criteria of PEN-FAST+ score.

This study was performed in three successive steps. In the first step, we applied the PEN-FAST score retrospectively in the latest 27 adult patients diagnosed in our department with a proven immediate or delayed allergy to penicillin through penicillin STs or positive drug provocation test (DPT). Fourteen and 13 patients had allergic HS proven by prick tests or intradermal tests positive for penicillin during immediate or delayed readings, respectively or positive DPT (Figure 1(A) and Supplementary Materials). No hypersensitivity phenotype that can be explored through the allergological explorations is excluded.

In the second step, among these 27 patients, we assessed the clinical features of patients with a proven penicillin allergy misclassified by the PEN-FAST score (i.e., having a positive ST or a positive DPT result while having a PEN-FAST score of <3) and we reviewed existing literature to identify potential additional criteria to better classify these allergic patients (Figure 1(B)).

A multivariable logistic regression was performed to identify factors associated with true allergy by using the 3 PEN-FAST criteria with these additional criteria to develop a new score (PEN-FAST+) in a monocentric prospective cohort of 252 successive patients (over a period of six consecutive months, excluding the 27 patients from step 1) with a suspicion of HS (immediate or delayed) to penicillin with STs and DPT.

The third step was the comparison of PEN-FAST and PEN-FAST+ scores in our prospective cohort of 252 patients. The PEN-FAST and PEN-FAST+ scores were used in this cohort, and a posteriori and diagnostic performance were calculated. The diagnostic performance of the 2 scores was assessed by comparing the area under the receiver operating characteristic curve (AUC) with the Delong Test (8). The calculation of the cutoff scores for the clinical decision rules PEN-FAST and PEN-FAST+ allowed for determining the statistical performance of the 2 tests using the Youden index. Statistical analysis are detailed in Supplementary Materials.

For all patients, the penicillin STs were conducted and interpreted according to the ENDA recommendations (for more details see Supplementary Materials).

This was an observational study, and patients' informed consent was obtained in accordance with local ethics committee requirements.

## Results

The PEN-FAST score showed limitations in predicting the relapse of immediate skin HS or delayed MPE, with 28.6 (*n* = 4) and 38.4% (*n* = 5) of patients misclassified, respectively (Figure 1(A)). None of the patients met the criteria for the two points related to anaphylaxis or angioedema or severe cutaneous adverse reactions. Their inclusion was based only on either a history of “Five years or less since reaction” or “Treatment required for reaction (including unknown)”.

After reviewing the medical records of the misclassified patients with PEN-FAST, we identified two potential additional criteria, occurring in all penicillin-allergic patients who were misclassified for each other (Figure 1(B) and Supplementary Table S1): skin rash lasting more than 7 days and immediate reaction occurring in less than 1 h (generalized or localized on the palmoplantar area or scalp itching/heat sensation occurring in the four patients misclassified by PEN-FAST). To improve the PEN-FAST NPV using these two additional criteria, we aimed to develop a new score, PEN-FAST+, and compare its diagnostic performance with that of PEN-FAST in a prospective validation cohort of 252 consecutive adult patients referred in our center for suspected penicillin HS. The multivariable model is presented in Table 1.

**Table 1:** Multivariable model with variables from PEN-FAST and two additional criteria for the PEN-FAST + clinical decision rule.

Variables	Multivariable analysis			Point score
	OR (95% CI)	*p*-value	*β* coefficient	
PEN-FAST Criteria				
Five years or less since reaction	3.50 (1.41–8.69)	0.007	1.25	**1**
Anaphylaxis or angioedema or severe cutaneous adverse reaction	3.07 (1.00–9.38)	0.04	1.12	**1**
Treatment required for reaction	0.86 (0.28–2.64)	0.80	−0.15	**–**
Additional criteria (PEN-FAST+)				
Skin rash lasting more than 7 days	8.47 (2.83–25.28)	0.0001	2.13	**2**
Immediate reaction occurring in less than 1 h	9.50 (3.11–28.98)	0.0001	2.25	**2**

The number of points assigned to each score variable was weighted proportionally to its β coefficient similarly to PEN-FAST study (3) by approximating the full number without decimal.

CI, confidence intervals; OR, odds-ratio.

In our model, the third criterion of PEN-FAST was no longer relevant (**T**reatment required for reaction, *p* = 0.80).

**Figure 2:**
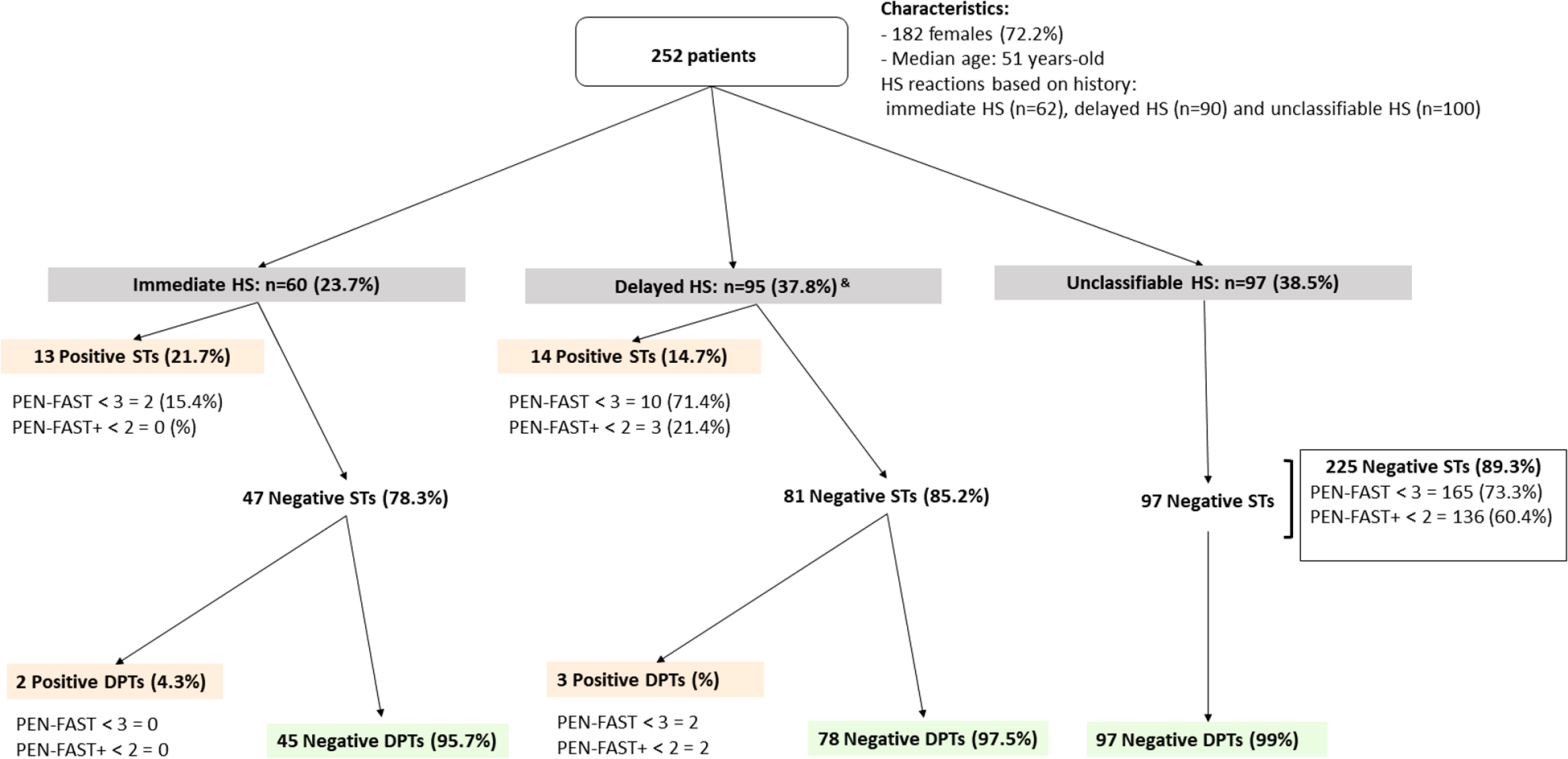
PEN-FAST and PEN-FAST+ score applied in a prospective cohort of 252 patients suspected to penicillin-allergy. HS, hypersensitivity; STs, skin tests, DTPs, drug provocation tests with penicillin. Orange: Penicillin-allergic patients, Green: non allergic patients. ^&^Four patients, two with an unclassifiable history of HS and two with an immediate history, had positive skin tests with delayed readings and were subsequently classified as having delayed HS. One patient with an unclassifiable history of hypersensitivity had a positive delayed drug provocation test and was also classified as having delayed HS.

Patient characteristics, allergy work-up, PEN-FAST, and PEN-FAST+ score results are shown in Figure 2. Briefly, STs were positive in 27/252 (10.7%) patients, 13 with immediate and 14 with delayed readings, who were confirmed to be allergic to penicillin. For 12 patients, the PEN-FAST score was <3 (44.4%): 2 and 10 with immediate and delayed HS, respectively. With PEN-FAST, 37% of patients (*n* = 10) with delayed allergic penicillin HS were misclassified. With PEN-FAST+, 3 patients with delayed HS confirmed by a ST (11.1%) were misclassified (Figure 2). Among them, 2 were correctly classified by PEN-FAST (less than 5 years since reaction and treatment received): one with benign MPE lasting less than 7 days and one with history of grade I HS but delayed positivity of STs controlled twice. None of them met the diagnostic criteria for DRESS syndrome. PEN-FAST+ correctly classified all penicillin allergic patients with immediate HS.

## Discussion

**Figure 3:**
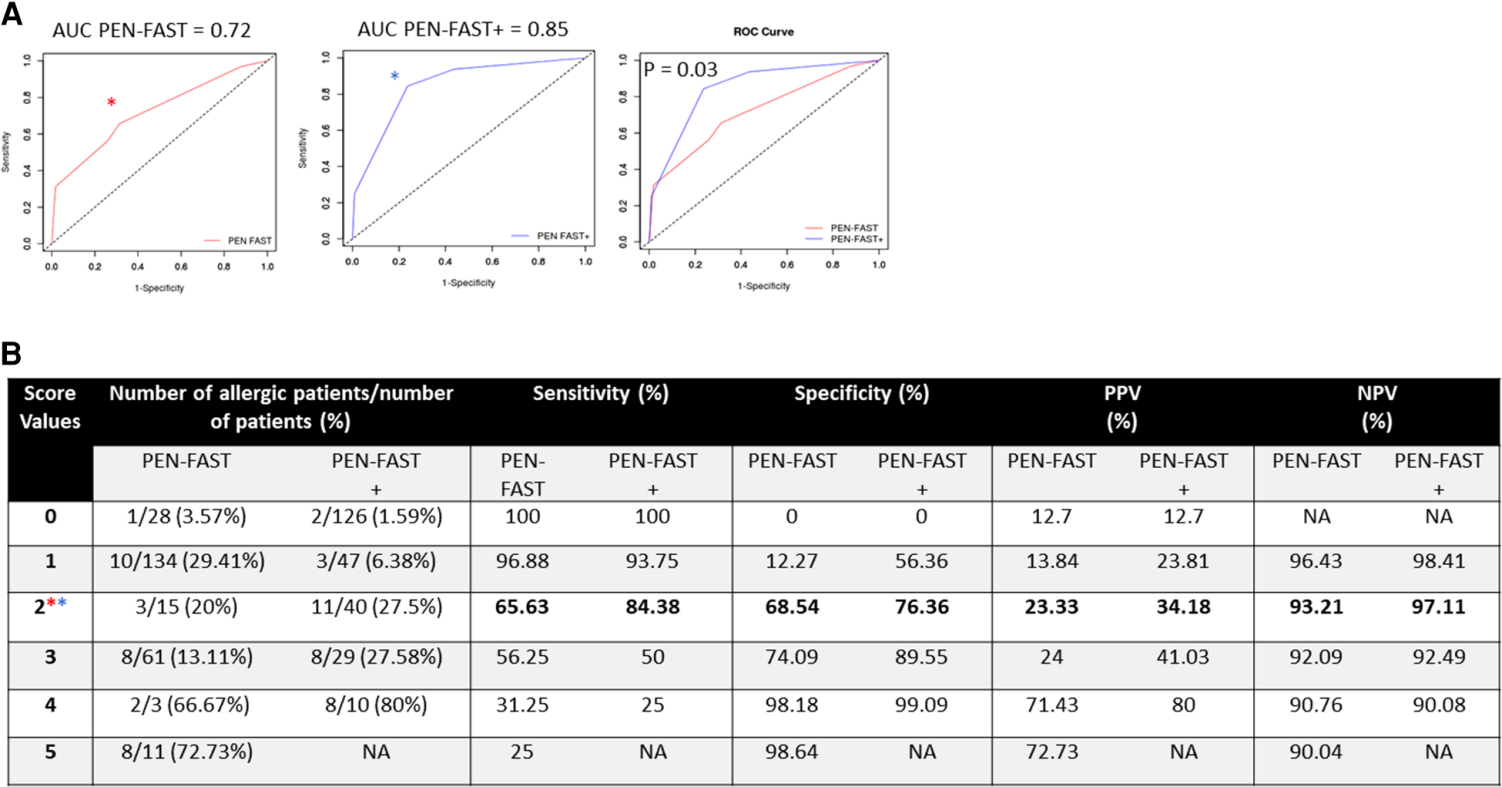
Diagnostic performances for PEN-FAST and PEN-FAST+. (**A**) Comparison of the AUC for PEN-FAST and PEN-FAST+. (**B**) Derivation of cutoff scores for clinical decision rules for PEN-FAST and PEN-FAST+, determining the statistical performance of the 2 tests using the Youden index: *Youden index PEN-FAST, *Youden index PEN-FAST+. AUC, area under the receiver operating characteristic curve; DPT, drug provocation test; HS, hypersensitivity; NA, not applicable; NPV, negative predictive value; PPV, positive predictive value; ST, skin test.

A total of 88.9% of patients with positive STs and 84.4% of truly allergic patients (shown by positive STs or DPT) were correctly identified by PEN-FAST+ compared with 55.6% and 56.2%, respectively, by PEN-FAST. Although we increased the sensitivity, we did not decrease the specificity. Indeed, compared with PEN-FAST, PEN-FAST+ proposed STs for 5 less non-allergic patients. The AUC was significantly higher for PEN-FAST+ than PEN-FAST (85% vs. 72%, *p* = 0.03) (Figure 3(A)). The threshold to identify patients with low-risk penicillin allergy who may not require an ST is defined here by the Youden index, with a PEN-FAST+ score <2 (Figure 3(B)).

Different penicillin-allergy decision clinical rules have been proposed, with a risk of misclassifying truly sensitized patients from 3.7% to 10% (2, 9–11).Unfortunately, regardless of the score, the objective of 100% NPV is difficult to reach because of memory bias. The effectiveness of the PEN FAST score was recently validated in a multicenter randomized clinical trial involving 351 participants with a low-risk immediate penicillin allergy (PEN-FAST score <3) (7). The results demonstrated that direct oral penicillin DPT was non-inferior to the current standard of STs followed by 1-step oral DPT if the ST results were negative. However, this study exclusively analyzed patients with immediate HS occurring up to one hour after penicillin DPT. Moreover, MPE, the most common form of penicillin allergy, was not clearly distinguished in previous studies evaluating penicillin-allergy decision rules (2, 7, 9–11).

Although it is a mild form of delayed HS in the majority of cases, some studies have demonstrated that prolonged MPE (lasting more than 5–7 days), sometimes with systemic symptoms, may be an early form of drug reaction with eosinophilia and systemic symptoms (DRESS), a severe cutaneous adverse drug reaction, with an overlap between these two forms (12–14). The definition of severe MPE (1) has been proposed by Romano et al, but identifying these cases can be challenging for non-drug allergist specialists. In such cases, there is a risk of developing DRESS on subsequent re-exposure to penicillin. Therefore, it is clinically highly relevant to use a score with the highest NPV to ensure the confidence of physicians and their adherence to the guidelines.

PEN-FAST has been validated in large cohorts of patients with history of penicillin allergy worldwide (2, 7, 9). The 3 criteria selected for its calculation are easy for patients to remember and can be easily retrieved by non-allergist doctors.

Furthermore, there is a risk of false positives with immediate reading of STs. Among the two patients with a positive ST and a PEN-FAST score of less than 3, the possibility of a false positive result for the ST cannot be ruled out.

## Conclusion

Our results suggest that the PEN-FAST+ score is a better tool for identifying penicillin-allergic patients, especially those with delayed HS with MPE lasting more than 7 days, which can be serious. A large prospective and multicentric study using PEN-FAST+ would be beneficial to better select patients at low risk of reaction, particularly those with delayed HS who could have a direct DPT without previous STs, for delabeling penicillin allergies.

## Data Availability

The raw data supporting the conclusions of this article will be made available by the authors, without undue reservation.
