# Supplementary material for: Assessing delayed penicillin hypersensitivity using the PENFAST+ score
Source: Front Allergy. 2023 Nov 13;4:1302567. doi: 10.3389/falgy.2023.1302567 (PMC10680989; doi:10.3389/falgy.2023.1302567)
Supplement: Supplementary file 1 [file Table1.pdf]

Supplemental

Patients and methods

Penicillin allergy explorations results were used as the gold standard to define a penicillin allergy. For each patient, results of penicillin allergy explorations were compared with those of PEN-FAST and PEN-FAST+ scores. This was an observational study, and patients' informed consent was obtained in accordance with local ethics committee requirements.

Our inclusion and exclusion criteria were the same than in the PEN-FAST study [2]: were included adult patients with history of reaction with one or more penicillin.

Were excluded drug reaction not classified in immediate and delayed HS and not explored with STs, e.g. immunological nephritis, drug hepatitis, serum sickness and isolated fever. We also excluded pregnancy and patients with isolated symptoms non evocative of HS (e.g., headache, candidiasis, isolated digestive disturbance).

Penicillin allergy exploration results were used as the gold standard to define a penicillin allergy.

Penicillin skin tests and penicillin provocation tests methods:

All patients were explored according to ENDA/EAACI recommendations (1). Briefly, all patients underwent skin prick tests, intradermal tests and/or patch tests with recommended dilutions and readings, with penicillin commercial solutions. The suspected penicillin was tested when the patient knew it. Amoxicillin-clavulanic acid was tested when the suspected penicillin was unknown. Skin prick tests were performed with undiluted commercial solution of penicillin: amoxicillin and amoxicillin-clavulanic acid (both 200 mg/ml). In case of severe immediate hypersensitivity (HS), more dilutions were used. Freshly prepared penicillin diluted with saline was used for intradermal skin tests with successive dilutions for amoxicillin and amoxicillin-clavulanic acid (0.2, 2 and 20 mg/ml). Skin prick and intradermal tests were performed on the volar forearm and read after 20 min, with saline and chlorhydrate histamine (10 mg/ml, Stallergenes, Montrouge, France) as negative and positive controls, respectively. Skin prick and intradermal test results were considered positive if the increase in the largest diameter of the wheal was ≥ 3 mm and it was surrounded by erythema. When a delayed reaction was suspected, patch tests with amoxicillin trihydrate (10% pet.) and clavulanic acid (10% pet.) (Chemotechnique, Velinge, Sweden) were performed and read between day 2 and day 5 according to the International Contact Dermatitis Research Group recommendations as previously

reported in drug HS. Reactions from + to +++ were considered positive. When a patch test was negative, skin prick and intradermal tests with readings at 20 min and day 2 were performed.

Penicillin oral provocation tests (DPTs) were performed with the suspected penicillin when the skin test result remained negative. The challenge was performed during a 1-day hospital stay, with strict supervision of blood pressure, heart rate, and oxygen saturation before, during and up to 2 hr after the challenge. This was a simple-blind placebo-controlled oral challenge; with at least 2 step doses of penicillin. The challenge was stopped when a clinical reaction occurred and was considered positive if saturation decreased < 90% and/or blood pressure decreased > 30% and/or a skin eruption (urticaria, angioedema or rash) occurred up to 7 days after the challenge.

PEN-FAST+ development and validation methods:

STEP 1: PEN-FAST retrospective evaluation in penicillin-allergic patients

We first analyzed PEN-FAST decision rules in a retrospective cohort of penicillin-allergic patients (immediate or delayed positive ST) (Supplemental Table 1). PEN-FAST results were incorrect in 4/14 (28.6%) of patients with immediate HS and 5/13 (38.4%) with delayed HS. The 4 patients with immediate HS with PEN-FAST score < 3 (low risk of penicillin HS) all had grade I HS with subjective symptoms such as pruritus or paresthesia or a heat feeling of the extremities within the first hour after penicillin intake. Concerning the 5 patients with delayed HS misclassified by PEN-FAST, 3 had MPE lasting more than 7 days, and one of them had fever, but none had criteria for DRESS syndrome.

STEP 2: Choice of 2 additional criteria for PEN-FAST+

We assessed the clinical features of patients with a proven penicillin allergy misclassified by the PEN-FAST score and we reviewed existing literature to identify potential additional criteria to better classify these allergic patients. We identified 2 potential additional criteria (PEN-FAST+): 1) skin rash lasting at least 7 days and 2) immediate reaction occurring in < 1 hr, with palmoplantar, genital, ear, and/or head, or generalized itching/tingling/heat feeling.

The 2 additional criteria were based on the 9 patients misclassified (5 with delayed hypersensitivity and 4 with immediate hypersensitivity) with the PEN-FAST and data from the literature (See Supplemental Table 1). In the case of the 5 delayed penicillin-allergic patients, all of them had a rash at least 7 days, and for the 4 immediate penicillin-allergic patients, all had reactions occurring within 1 hour after drug intake. The chronology of symptom onset was considered an item discriminating immediate and non-immediate reactions to penicillin. The duration of the reaction at least 7 days was also considered because it suggested possible moderate-to-severe maculopapular exanthema.

STEP 3: PEN-FAST and PEN-FAST+ comparison using a prospective validation cohort of suspected penicillin allergic patients

To evaluate PEN-FAST and PEN-FAST+ scores, we prospectively included all patients who attended our hospital for either a penicillin DPT (amoxicillin or amoxicillin-clavulanic acid) after a negative ST or a cefixime/ceftriaxone DPT in substitution in case of penicillin allergy (positive ST to a penicillin) between February and August 2021. We consecutively included 252 adults.

Statistical analysis:

We used multivariable logistic regression to identify factors associated with true allergy by using the 3 PEN-FAST criteria with our 2 additional criteria (Table 1). The number of points assigned to each score variable was weighted proportionally to its β coefficient by approximating the decimal points to the nearest unit, similar to the PEN-FAST study2. The diagnostic performance of the 2 scores was assessed by comparing the area under the receiver operating characteristic curve (AUC) with the Delong Test8. The calculation of the cutoff scores for the clinical decision rules PEN-FAST and PEN-FAST+ allowed for determining the statistical performance of the 2 tests using the Youden index.

The specificity was defined as the probability that a non-allergic patient was classified at low risk of allergy with PEN-FAST or PEN-FAST+ score < 3. The sensitivity was defined as the probability that an allergic patient was classified at risk of allergy (PEN-FAST score ≥ 3). The negative predictive value (NPV) was defined as the probability that a patient classified at low risk of allergy was non-allergic and the positive predictive value (PPV) as the probability that a patient classified at high risk of allergy was truly allergic to penicillin. Statistical analyses were performed using JMP v17, SAS Institute.

Patient number	Age (years-old)/ Sex	Hypersensitivity reaction	Reaction latency (years)	Time of onset	Duration of the reaction	Culprit drug	Treatment of the reaction	Skin test positivity	PEN-FAST Score
**Delayed penicillin-hypersensitivity reactions**									
1	50/M	MPE	3	2 days	7 days	amoxicillin-clavulanic acid	antihistamines, topical steroid	delayed reading	3/5
2	32/F	Flexural MPE with fever	2	1 day	6 days	amoxicillin-clavulanic acid	topical steroid	no, positivity of drug provocation test	3/5
3	49/F	Diffuse MPE (more than 80% of body surface area with lymphopenia)	2	7 days	11 days	amoxicillin-clavulanic acid	topical steroid	delayed reading	3/5
4[1]	51/M	diffuse MPE and facial oedema	15	1 day	7 days	amoxicillin-clavulanic acid	oral steroid	delayed reading	1/5
5[1]	72/F	MPE	0.5	10 days	30 days	amoxicillin-clavulanic acid	topical steroid	delayed reading	1/5
6	73/M	diffuse MPE	3	1 day	7 days	amoxicillin	antihistamines	delayed reading	3/5
7[1]	62/F	MPE	7	few days	8 days	amoxicillin	antihistamines oral steroid	delayed reading	1/5
8	31/F	MPE	2	1 day	5 days	amoxicillin	NK	delayed reading	5/5
9[1]	25/F	diffuse MPE	14	few days	7 days	amoxicillin	antihistamines, topical steroid	delayed reading	1/5
10	73/M	PEAG/DRESS	3	3 days	6 weeks	amoxicillin	topical steroid	delayed reading	5/5
11[1]	20/M	MPE and palmoplantar oedema	50	7 days	8 days	Penicillin G	NK	delayed reading	1/5
12	19/F	diffuse MPE and facial oedema with eosinophilia	3	3 days	4 days	amoxicillin	NK	delayed reading	3/5
13	18/F	MPE	3	2 days	1 day	amoxicillin-clavulanic acid	NK	delayed reading	3/5
**Immediate penicillin-hypersensitivity reactions**									
14	35/F	generalized urticaria, dyspnea, diarrhea, hypotension	3	20 min	less than 1 day	amoxicillin	Saline expansion	immediate reading	4/5
15	31/M	generalized urticaria, loss of consciousness, hypotension	3	15 min	less than 1 day	amoxicillin	systemic steroid, saline expansion antihistamines	immediate reading	5/5
16[1]	34/M	generalized urticaria	4	less than 1 hour	few hours	amoxicillin	systemic steroid	immediate reading	2/5

17[1]	57/M	generalized urticaria and pruritus	12	10 min	less than 1 day	penicillin (NK)	NK	immediate reading	0/5
18	77/M	generalized urticaria, lips oedema, pruritus, dyspnea, hypotension	2	few min	less than 1 day	amoxicillin	oxygen therapy, adrenaline	immediate reading	5/5
19	80/M	diffuse rash	2	3 days	7 days	amoxicillin	topical steroid	immediate reading	3/5
20[1]	73/F	itching sensation of fingers, hand redness, dizziness with transient loss of consciousness	3	few minutes	less than 1 day	amoxicillin	no	immediate reading	2/5
21	24/M	urticaria and palmoplantar pruritus	3	less than 1 hour	less than 1 day	amoxicillin	anthistamines	immediate reading	3/5
22[1]	36/F	urticaria and facial redness and angioedema	2	20-30 min	30 min	amoxicillin	no	immediate reading	2/5
23	43/F	generalized urticaria, hand oedema and hypotension	3	20 min	less than 1 day	amoxicillin-clavulanic acid	systemic steroid, saline expansion, antihistamines	immediate reading	5/5
24	58/F	generalized urticaria, palmoplantar oedema, dyspnea	3	15 min	less than 1 day	amoxicillin	adrenaline, anthistamines	immediate reading	5/5
25	36/F	generalized urticaria, pruritus, dizziness	2	10 min	less than 1 day	amoxicillin	NK	immediate reading	3/5
26	58/M	diffuse urticaria and rash, loss of consciousness	3	30 min	less than 1 day	amoxicillin-clavulanic acid	NK	immediate reading	3/5
27	40/F	generalized urticaria, facial oedema, hypotension	2	30 min	less than 1 day	amoxicillin	adrenaline, systemic steroid, antihistamines	immediate reading	5/5

Supplemental Table 1. Clinical characteristics of the 27 patients in the retrospective cohort during the step 1.
Abbreviations: DRESS, drug reaction with eosinophilia and systemic symptoms; F, female; M, male; min, minutes; MPE, maculopapular exanthema; NK, not known; SDRIFE, symmetrical drug-related intertriginous and flexural exanthema.

[1] *The PEN-FAST score was <3 and misclassified 4 immediate and 5 delayed penicillin-allergic patients, respectively.*
